# Radical *Versus* Non-Radical Resection for Early-Stage Retroperitoneal Sarcoma: A Propensity Score-Matched Analysis

**DOI:** 10.3389/fonc.2021.706543

**Published:** 2021-07-14

**Authors:** Chengxin Weng, Jiarong Wang, Jichun Zhao, Ding Yuan, Bin Huang, Tiehao Wang

**Affiliations:** ^1^ Department of Vascular Surgery, West China Hospital, Sichuan University, Chengdu, China; ^2^ West China School of Medicine, West China Hospital, Sichuan University, Chengdu, China

**Keywords:** retroperitoneal sarcoma, radical resection, propensity score, radiotherapy, early stage

## Abstract

**Background:**

The appropriate surgical procedure for early-stage retroperitoneal sarcoma (RPS) is unclear. Thus, we used a national database to compare the outcomes of radical and non-radical resection in patients with early stage RPS.

**Methods:**

This retrospective study included 886 stage I RPS patients from 2004 to 2015 in the SEER database. Outcomes were compared using the multivariate Cox proportional hazards models and the results were presented as adjusted hazards ratio (AHR) with corresponding 95% confidence intervals (95%CIs). Propensity score-matched analyses were also performed for sensitive analyses.

**Results:**

For the 886 stage I RPS patients, 316 underwent radical resection, and 570 underwent non-radical resection, with a median follow-up of 4.58 (2.73-8.35) years. No difference was observed in overall mortality (AHR 0.84, 95%CI 0.62-1.15; P = 0.28) or RPS-specific mortality (AHR 0.88, 95%CI 0.57-1.36; P = 0.56) between groups. The results were similar in propensity score-matching analyses. However, subgroup analysis revealed that radical resection was associated with significantly decreased risks of overall mortality in male (AHR 0.61, 95%CI 0.38-0.98; P = 0.04) and in patients with radiotherapy (AHR 0.56, 95%CI 0.32-0.98; P = 0.04).

**Conclusion:**

Radical resection did not improve midterm survival outcomes compared with non-radical resection in overall patients with early stage RPS. However, male patients or patients who received radiotherapy might benefit from radical resection with improved overall survival.

## Introduction

Retroperitoneal sarcomas (RPSs) are rare types of sarcomas arising from the retroperitoneum, which represent approximately 15% of all soft tissue sarcomas (1). The estimated incidence less than 1 case per 100,000 inhabitants/year, with nearly 4,500 new cases diagnosed yearly in Europe and 1785 in the United States (2–4). The long-term prognosis of RPSs varies the between subtypes but is always relatively poor, with a 5-year overall survival of approximately 50% (5). Due to poor responses to systemic therapies, surgery has long been the mainstay of treatment for RPS (5–7). However, the large size of the tumors, their adjacent relationship to vital structures, and the propensity of local recurrence render surgical procedures challenging and complicated.

Over the recent decade, many centers proposed a radical surgical strategy involving *en bloc* resections of the tumors with adjacent organs or structures to achieve maximum R0 resection and minimize the risk of local recurrence (8, 9). In practice, surrounding structures (e.g. psoas, kidney, or part of the colon), were also excised with the mass even when not infiltrated. However, these radical resections may increase the risk of major postoperative morbidity and affect the quality of life in high-risk patients (10). Additionally, a recent study reported that the number of organs excised could independently predict worse long-term overall survival (11). Therefore, the Transatlantic RPS Working Group has advocated establishing a stage/histological-specific and data-driven standard of surgical strategy for a selective organ resection in PRS patients (12). Specifically, the impact of radical resection in patients with early-stage RPS stratified by different histology has not been investigated before. To fully address the confounding factors that may affect survival outcomes, we performed a propensity score matching analysis using the Surveillance, Epidemiology, and End Results (SEER) registry to evaluate the role of radical resection in early-stage RPS.

## Methods

### Data Sources

The SEER program collected cancer data from population-based cancer registries covering about 35% (https://seer.cancer.gov/about/overview.html) of the United States population including various races since January 1, 1973. We extracted the dataset from the SEER*Stat Database ‘Incidence -SEER 18 Regs Custom Data (with additional treatment fields) based on the November 2017 submission (1973-2015 varying)’. We signed a data-use agreement and accessed the SEER dataset through the ID 10672-Nov2018. The study used de-identified data and did not require ethical approval. The data-use agreement is in the **Supplementary materials_1**.

### Study Population

Our retrospective cohort study included patients with American Joint Committee on Cancer (AJCC) Stage I retroperitoneal sarcomas who underwent surgery between January 2004 and December 2015. The patients were identified by cross-referencing anatomical sites and histology. They were included when they displayed AJCC Stage I tumors originating primarily in the retroperitoneum (ICD-O-3 Site code:C480) and with the ‘common sarcomas of retroperitoneum’ histology code as defined by the *AJCC Cancer Staging Manual, Eighth Edition* (13). We considered the patients displaying both 6^th^ and 7^th^ AJCC staging and included patients with 7^th^ AJCC staging when we found differences between the 6^th^ and 7^th^ editions. The patients who underwent cancer-directed surgery were selected and divided into a radical resection group and a non-radical resection group according to the extent of surgery.

Generally, retroperitoneal sarcomas are quite large, present multiple structures, and are prone to recurrence. To maximize the achievement of R0 resections, the current guidelines recommend a radical surgical strategy involving the resection of the tumor with its adjacent organs or structures to minimize the risk of local recurrence (14). In the SEER database, a radical resection was defined as a ‘partial or total removal of the primary site WITH an *en bloc* resection (partial or total removal) of other organs (code 60 from both 1983 to 1997 and 1998 +)’. Partial removal of the primary site can be considered as a radical surgery if vital organs are involved. Moreover, it was difficult to obtain a reliable microscopic evaluation of the margins even with postoperative pathology due to an incomplete margin sampling and difficulties to determine the primary site in the clinical practice. The other surgery procedures were defined as non-radical resection, including the surgeries considered as “debulking”. The following patients were excluded: patients with incompetent information on tumor size and grade and with recurrent tumors or multiple primary tumors.

### Variables and Outcomes of Interest

The primary endpoint of our study was overall mortality after surgery and the secondary outcome was RPS-specific death after surgery. The survival time was defined as the time between the date of RPS diagnosis and death or it was right-censored at the follow-up cutoff (November 2017). Notably, we found crosses in the survival curves around 8 years in our exploratory analyses. Landmark analyses were performed to estimate outcomes within and beyond 8 years (i.e., the landmark point).

The primary independent variable of interest was surgical procedures (radical *vs* non-radical). The other covariates of interest, including the age at diagnosis, gender, histology, tumor size, tumor grade, and radiotherapy, are described in **Supplementary materials_2**.

### Statistical Analysis

#### Baseline

The distributions of the baseline characteristics were described as medians with interquartile ranges (IQRs) for the continuous variables or as percentages for the categorical variables. We compared the different groups using logistic regression models for all variables.

#### Cox Proportional Hazards Regression for Effect Evaluation

The effect of radical resection compared with non-radical resection was calculated using the Cox proportional hazards regression model. Age, gender, histology, tumor grade, and treatment with radiotherapy were adjusted because these variables were considered clinically significant.

Other variables that changed the hazards ratio by at least 10 percent when added to the univariate model or deleted from the model, were also used in the final model. The results are presented as adjusted hazards ratios (AHR) with a corresponding 95% confidence interval (95%CI).

#### Sensitivity and Subgroup Analyses

Propensity score matching analyses were used for sensitivity analysis. First, we used a propensity score matching was used to minimize the differences between the groups at baseline. The propensity score for the surgery procedure was evaluated using logistic regression based on the following variables: age, gender, histology, tumor size, tumor grade, radiotherapy, and chemotherapy. Matching ratios of 1:1 was were used in the final analysis. We performed The Cox proportional hazards regression models adjusted by the propensity scores using the matched cohort were performed to re-evaluated the effect of radical resection and further control the potential confounders.

TIn order to explore the potential heterogeneity among between different populations, we performed multivariate subgroup analyses were performed stratified by age (≤ 60 and > 60 years), gender, tumor size (≤150mm and >150mm) (15), tumor grade (Grade I and Grade II-IV), and radiotherapy. We calculated the P values to evaluate of the interactions between the surgery procedure and each subgroup variable were evaluated using likelihood ratio tests by including the interaction terms in the Cox regression model.

TAll he statistical analyses were performed using R studio Version 1.2.1335. TAllhe P values were 2-sided, with a significance level of 0.05.

## Results

### Baseline Characteristics

**Figure 1:**
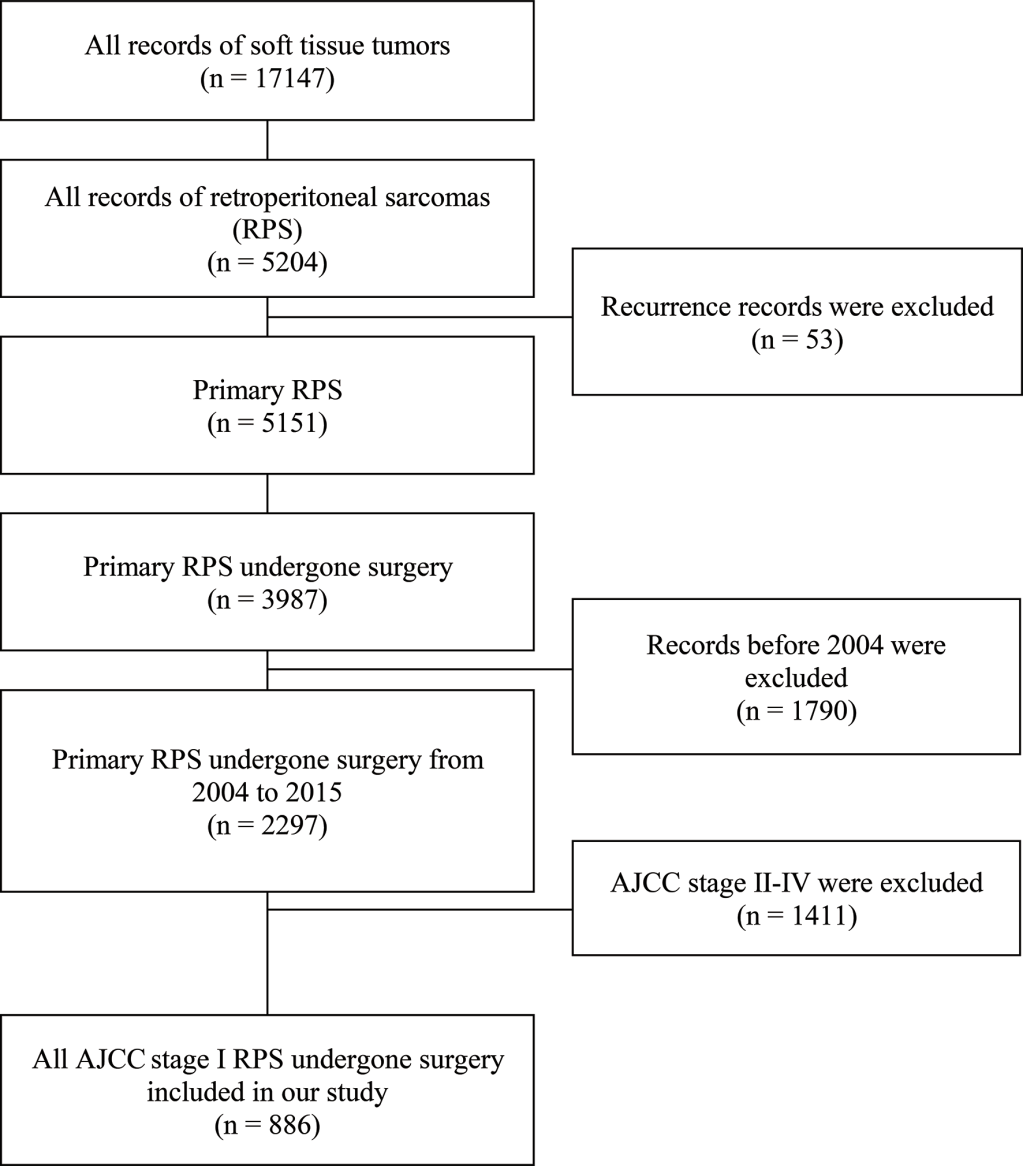
Flow diagram of patient selection from the Surveillance, Epidemiology, and End Results (SEER) database.

We identified a total of 886 patients who underwent effective surgical resection. The patient selection process is shown in **Figure 1**. A total of 316 (35.7%) patients underwent radical resections and the other 570 (64.3%) underwent non-radical resections. The patients who underwent radical resections of retroperitoneal sarcoma were younger than those who received non-radical resections [60(51-69) *vs* 63(53-72) years; *P* = 0.01]. They also displayed a larger tumor size [222/316(70.3%) displayed tumors >150 mm compared with 283/570(49.6%); *P* < 0.001]. Additionally, the patients diagnosed with stage IA [59(7.1%)] retroperitoneal sarcomas were rare in this study. The detailed baseline characteristics for the overall population are shown in **Table 1**.

**Table 1:** Baseline characteristics of patients who received radical resection *versus* non-radical resection before and after propensity score matching (PSM).

Variables	Before PSM^a^			After PSM^a^		
	Non-radical (N=570)	Radical (N=316)	*P Value*	Non-radical (N=287)	Radical (N=287)	*P Value*
**Median age, yr (IQR)**	63 (53-72)	60 (51-69)	0.014*	61 (51-71)	60 (51-68)	0.608
**Sex, n (%)**			0.507			0.738
Female	297 (52.1)	172 (54.4)		159 (55.4)	155 (54.0)	
Male	273 (47.9)	144 (45.6)		128 (44.6)	132 (46.0)	
**Histology, n (%)**			<0.001*			0.683
Leiomyosarcoma	94 (16.5)	33 (10.4)		29 (10.1)	26 (9.1)	
Liposarcoma DD^b^	43 (7.5)	60 (19.0)		33 (11.5)	48 (16.7)	
Liposarcoma WD^c^	247 (43.3)	153 (48.4)		164 (57.1)	151 (52.6)	
Malignant fibrous histiocytoma (MFH)	2 (0.4)	1 (0.3)		0 (0)	1 (0.3)	
Other sarcomas	186 (32.3)	70 (21.8)		61 (21.3)	62 (21.6)	
**Stage**			0.002*			0.229
IA	49 (8.6)	10 (3.2)		16 (5.6)	10 (3.5)	
IB	521 (91.4)	306 (96.8)		271 (94.4)	277 (96.5)	
**Tumor size, n (%)**			<0.001*			0.782
≤150 mm	261 (48.0)	91 (29.1)		80 (27.9)	83 (28.9)	
>150 mm	283 (52.0)	222 (70.9)		207 (72.1)	204 (71.1)	
**Grade, n (%)**			0.718			0.769
Grade I	393 (76.9)	219 (75.8)		220 (76.7)	217 (75.6)	
Grade II-IV	118 (23.1)	70 (24.2)		67 (23.3)	70 (24.4)	
**Radiotherapy, n (%)**			0.410			0.775
No	431 (75.6)	231 (73.1)		215 (74.9)	212 (73.9)	
Yes	139 (24.4)	85 (26.9)		72 (25.1)	75 (26.1)	
**Chemotherapy, n (%)**			0.312			1.000
No/Unknown	543 (95.3)	296 (93.7)		273 (95.1)	273 (95.1)	
Yes	27 (4.7)	20 (6.3)		14 (4.9)	14 (4.9)	

Data were expressed as median (interquartile range, IQR); n (%), percentages were calculated after excluding missing cases from the denominator. ^a^PSM, propensity score matching; ^b^DD, dedifferentiated; ^c^WD, well-differentiated; ^*^statistically significant.

### Survival Outcomes

For the 886 patients identified with stage I RPS, the median follow-up evaluated by the reverse Kaplan Meier method was 4.58 years (interquartile range 2.73-8.35 years). During the follow-up period, a total of 73 (23.1%) deaths were observed in the radical resection group and 158 (27.7%) in the non-radical resection group. The 1, 3, 5 and 10-year overall survival rates were 93.6%, 85.9%, 75.4%, and 50.9% in the radical resection group *versus* 94.2%, 82.1%, 71.0%, and 52.1% in the non-radical resection group, respectively. RPS-specific deaths occurred in 38 (12.0%) patients in the radical resection group and 76 (13.3%) in the non-radical resection group. The 1, 3, 5, and 10-year RPS-specific survival rates were 97.3%, 91.3%, 85.3%, and 85.3% in the radical resection group *versus* 97.2%, 91.3%, 84.7%, and 69.3% in the non-radical resection group.

**Figure 2:**
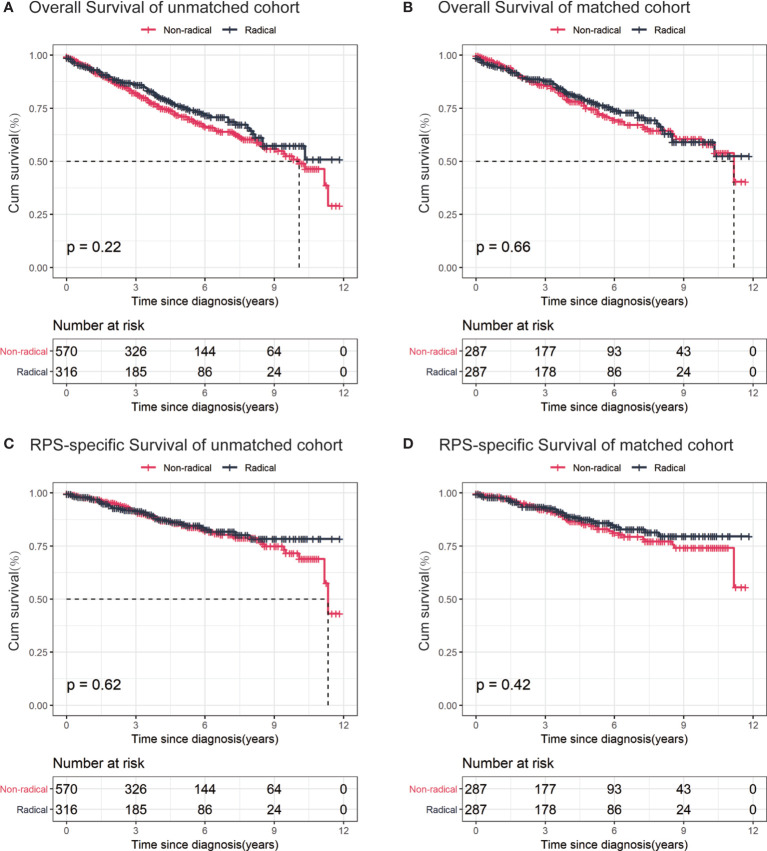
Kaplan–Meier curves for stage I retroperitoneal sarcoma (RPS) patients. **(A)** overall survival of unmatched cohort; **(B)** overall survival of propensity score-matched cohort; **(C)** RPS-specific survival of unmatched cohort; **(D)** RPS-specific survival of propensity score-matched cohort.

The Kaplan-Meier curves of the overall and RPS-specific mortality in the overall and PSM-matched cohorts are shown in **Figure 2**. In our landmark analysis, we found that the overall (*P* = 0.23) and the RPS-specific mortality (*P* = .98) did not differ significantly between the groups within 8 years after surgery. However, 8 years after surgery, the RPS-specific mortality was higher in the non-radical resection group than the radical resection group numerically but non-significantly (*P* = 0.05). The details are shown in **Supplementary Figure 1**.

### Univariate and Multivariate Analyses

To estimate the effect of the surgery modification on the overall and RPS-specific mortality, we performed univariate and multivariate-adjusted Cox analyses in the unmatched cohort. We did not observe any significant difference between radical and non-radical resections in terms of overall mortality in the univariate analysis (HR 0.79, 95%CI 0.59-1.06; *P* = 0.12). The results were similar in the basic and fully adjusted models (**Table 2**). As for the RPS-specific mortality, we also did not observe any significant difference between radical and non-radical resections in both the univariate analysis (HR 0.86, 95%CI 0.57-1.30; *P* = 0.48) and the multivariate analysis (AHR 0.88, 95%CI 0.57-1.36; *P* = 0.56).

**Table 2:** Hazard ratios (HR) of overall mortality and RPS-specific mortality.

Model	Overall mortality		RPS-specific mortality	
	HR (95% CI)	*P Value*	HR (95% CI)	*P Value*
Unmatched cohorts				
Univariate	0.79 (0.59-1.06)	0.12	0.86 (0.57-1.30)	0.48
Basic	0.85 (0.63-1.15)	0.30	0.90 (0.58-1.40)	0.65
Full	0.84 (0.62-1.15)	0.28	0.88 (0.57-1.36)	0.56
Matched cohorts				
PS matching	0.93 (0.66-1.30)	0.66	0.83 (0.53-1.31)	0.42
PS adjusted	0.94 (0.67-1.31)	0.70	0.83 (0.52-1.31)	0.42

Cox regression estimated the effect of radical resection versus non-radical resection on stage I retroperitoneal sarcoma (RPS) patients; P value < 0.05 is regarded as statistically significant. HR > 1 is associated with worse overall survival or RPS-specific survival; HR < 1 is associated with improved overall survival or RPS-specific survival. Basic model was adjusted by age, gender, histology, and grade. Full model was adjusted by age, gender, histology, grade, tumor size, and radiotherapy. Propensity score (PS) was estimated using logistic regression based on the following variables: age, gender, histology, tumor size, grade, radiotherapy, and chemotherapy. PS adjusted model was adjusted by propensity score after matching.

### Propensity Score Analyses

The propensity-score analyses generated 287 matched pairs, i.e., a total of 574 patients with similar baseline characteristics and propensity to receive radical and non-radical resections (**Table 1**).

In the matched cohort, a total of 64 (22.3%) deaths were observed in the radical resection group and 74 (25.8%) in the non-radical resection group. RPS-specific deaths occurred in 33 (11.5%) patients in the radical resection group and 42 (14.6%) in the non-radical resection group. We did not observe any differences in overall (HR 0.93, 95%CI 0.66-1.30; *P* = 0.66) or RPS-specific mortality (HR 0.83, 95%CI 0.53-1.31; *P* = 0.42) between the groups in the matched cohort. After adjusting by propensity score, the effect of surgery modification on overall (AHR 0.94, 95%CI 0.67–1.31; *P* = 0.70) and RPS-specific mortality (AHR 0.83, 95%CI 0.52-1.31; *P* = 0.42) remained similar between the two groups, which was consistent with the results of the multivariate models (**Table 2**).

### Subgroup Analyses

**Figure 3:**
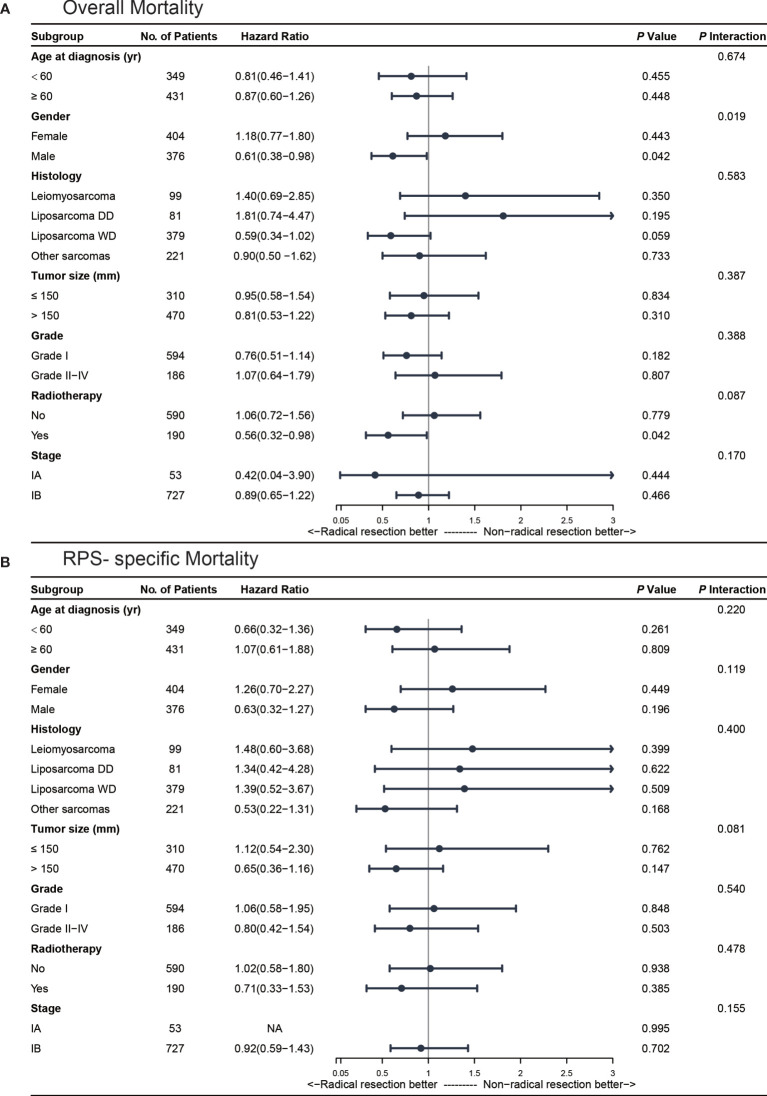
Multivariate subgroup analyses for stage I retroperitoneal sarcoma (RPS) patients. **(A)** subgroup analysis for overall mortality; **(B)** subgroup analysis for RPS-specific mortality.

To further address whether the effect of surgery modification differed in different groups of the population, we performed subgroup analyses according to age, gender, histology, tumor grade, tumor size, and radiotherapy. The results are summarized in **Figure 3** and **Supplementary Figure 2**.

#### Gender

The Kaplan-Meier curves of the overall survival stratified by gender are shown in **Supplementary Figure 3A**. In the subgroup of male patients (n = 376, 48.2%), radical resections were associated with a significantly decreased overall mortality (AHR 0.61, 95%CI 0.38-0.98, *P* = 0.04). After propensity score matching, the result was similar in male patients (HR 0.60, 95%CI 0.37-0.97; *P* = 0.04). Additionally, we found a significant association between gender and surgical procedure after adjusting for age, gender, histology, tumor size, tumor grade, and radiotherapy (*P_interaction_*= 0.02). As for RPS-specific mortality, we did not find any significant difference in the male patients who received radical or non-radical resections (AHR 0.63, 95%CI 0.32-1.27, *P* = 0.20) in our multivariate model or after propensity score matching (HR 0.63, 95%CI 0.32-1.25; *P* = 0.19).

#### Radiotherapy

A total of 190 (24.4%) stage I RPS patients received radiotherapy in the perioperative period. In our univariate model, radical resections were associated with a significantly decreased overall mortality (HR 0.52, 95%CI 0.30-0.91; *P* = 0.02) in the patients who received perioperative radiotherapy (**Supplementary Figure 3B**). After adjusting by confounding factors, radical resections with radiotherapy were also associated with a significantly decreased overall mortality (AHR 0.56, 95%CI 0.32-0.98; *P* = 0.04) and the results were similar after propensity score matching (HR 0.53, 95%CI 0.29-0.97; *P* = 0.04). As for the RPS-specific mortality, we did not find any significant difference between radical and non-radical resections in the patients who receive perioperative radiotherapy in our multivariate model (AHR 0.71, 95%CI 0.33-1.53, *P* = 0.39).

#### Histology

The patients were divided into four subgroups according to their histology as previously described. Nearly half of the patients [379 (48.6%)] were diagnosed with well-differentiated liposarcoma and 99 (12.7%) with leiomyosarcoma. The Kaplan-Meier curves of overall survival stratified by major histological patterns are shown in **Supplementary Figure 3C**. In our univariate model, radical resections were not associated with reduced overall mortality (HR 0.64, 95%CI 0.37-1.09; *P* = 0.10) or RPS-specific mortality (HR 1.49, 95%CI 0.59-3.75; *P* = 0.40) in the well-differentiated liposarcoma cohort. We found similar results in patients with leiomyosarcoma. For the patients with well-differentiated liposarcoma who received radical resections, the rate of overall mortality during the follow-up period was reduced numerically but not significantly with an absolute risk reduction of 7.1% (AHR 0.59, 95%CI 0.34-1.02; *P* = 0.06) after adjusting in the multivariate model.

## Discussion

The role of radical resections in RPS has been largely discussed in the recent decade and most studies advocate radical resections as the frontline surgical strategy due to a better local control (8–10). However, due to the limited sample size, only a few studies focused on the necessity of radical resections exclusively in stage I RPS. In this study, we reviewed stage I RPS patients who underwent radical or non-radical resections between 2004 and 2015 in the SEER national database. We found that the proportion of radical resections *versus* non-radical resections was close to 1:2, namely in most cases, the surgeons performed non-radical resections in early-stage RPS. Our results provided clinical evidence showing that radical resections did not improve survival outcomes compared with non-radical resections in overall stage I RPS patients in the mid-term follow-up. However, we also found that some groups of patients might benefit from radical resections in terms of overall survival, for instance, for male patients or patients who underwent perioperative radiotherapy.

Compared to previous studies assessing the role of radical resections, our study had two major improvements in methodology. First, nearly all the published studies failed to fully adjust for patient characteristics, tumor behavior, and adjuvant therapeutic strategy. Confounding factors, such as age, tumor histology, size, grade, radiotherapy, and chemotherapy, can affect long-term survival outcomes and cause bias. To fully address this issue, we also performed propensity score matching and conducted multivariate analysis adjusted for propensity scores. Secondly, as most of RPSs are initially diagnosed at an advanced stage, the evidence on the management of early-stage RPS was scarce and mainly deduced from advanced-stage tumors or subgroup analysis with a small sample size (5). Our study reviewed the SEER national database and retrieved 886 eligible patients, which may represent the largest cohort for stage I RPS patients. With these two improvements, we identified several noteworthy findings.

First, our subgroup analysis by gender in the two cohorts both suggested that male patients with stage I RPS may benefit from radical resections to improve their overall mortality. Furthermore, radical resections were still advantageous in male patients after accounting for the effects of age, histology, grade, tumor size, and radiotherapy. The improved overall mortality in male patients was also noted in a European study including RPS of all stages (15). A possible explanation for this gender-based disparity might be attributed to innate pelvic anatomic differences between males and females. In most circumstances, radical resections would include more organs in female patients than in males. Additionally, previous evidence revealed that the number of resected organs was adversely associated with long-term overall survival, which might explain the better overall survival in male patients after radical surgery (12). Besides gender differences, other factors, such as social characteristics (16), might also affect the rate of mortality and need more exploration in the future.

Second, our stratification analysis using different histological patterns did not reveal any significant difference in survival outcomes between the two groups. However, we noticed some discrepancies. We observed a numerically higher overall mortality in leiomyosarcoma patients after radical resections but it was lower in well-differentiated liposarcoma patients. To our knowledge, retroperitoneal sarcomas contain various pathologies, each characterized by distinct biological behaviors. It was reported (17) that well-differentiated liposarcomas tend to display local recurrence, while leiomyosarcomas show higher risks of distant recurrence. This might explain the different effects of radical resections in these two types of RPS. Future studies are warranted to further investigate this issue.

Third, we found that stage I RPS patients with perioperative radiotherapy were associated with reduced overall mortality after radical resections. About one-fourth (190/780) of the patients received radiotherapy in our study cohort and only 39.4% (75/190) received radical resections. National data showed that radical resections combined with radiotherapy were only adopted in a minority of stage I RPS patients. Due to a potential increase in overall mortality and long-term dysfunctions (18), determining the impact of radical resections and radiotherapy in combination is crucial (19). Given its potential toxicity and poor evidence, radiotherapy shows a narrow indication in RPS-related guidelines (20). Recently, Nussbaum (21) suggested that both pre- and post-operative radiotherapy could improve overall survival in RPS patients compared with surgery alone using the National Cancer Data Base. Though radiotherapy and radical resections were both associated with good local control effects (22, 23), there were concerns about the adverse quality of life outcomes when using both strategies. Our study showed that using radical resections and radiotherapy in early-stage RPS patients was relatively safe.

Fourth, we did not observe survival benefits after radical resections in stage I RPS patients, however, we visually observed a landmark point in the Kaplan-Meier curve approximately 8 years after surgery. Therefore, we performed a landmark analysis to further explore the presence of a time-point that could distinguish survival differences. Our analyses showed the superiority of radical resections compared with non-radical resections in overall and disease-specific survival after 8 years. However, the difference was only numerical but non-significant, which might be due to insufficient sample size and limited follow-up time. It is possible that the potential benefit of radical resections might emerge after 8 years or later. The French Sarcoma Group reported that 9.3% of patients with RPS experienced delayed (later than 5 years after surgery) local recurrences (24). Therefore, the traditional 5-year primary endpoint assessment for RPS patients might be insufficient to determine which therapeutic strategy could achieve the best long-term prognosis. Additionally, several pathological studies also observed histopathologic organ invasion in more than one-fourth of adherent organs even when it was not suspected intraoperatively (12). Although these pathological findings need to be confirmed in early-stage RPS, our study showed that clinicians should include 10-year long-term prognosis for early-stage RPS patients.

Despite several improvements in the methodology, our study was not devoid of limitations. First, the SEER database did not provide information on postoperative comorbidities and tumor recurrence. Hence, we were unable to systemically evaluate the safety of radical resections in early-stage RPS patients. Instead, our results on disease-specific deaths might shed light on tumor-related adverse outcomes. Second, as the onset of RPS is usually insidious, only a few patients diagnosed with RPS display a tumor size lower than 5cm. Hence, it is difficult to analyze the survival outcomes for patients with stage IA RPS. The surgery of patients with large tumors is usually associated with higher difficulties and risks. Successful R0/R1 resections during the initial treatment seem to influence survival outcomes. The resection margins and numbers of resected organs were, however, not available in the SEER database. Tumor size alone might only act as a moderate predictor of adverse outcomes in RPS but tumor grades and resection margins are believed to be stronger predicted factors (25). Additionally, the anatomic constraints and the high vascularization in the retroperitoneal space limit the possibility to achieve R0/R1 resections in many patients. These variables should also be considered as confounding factors for prognosis in future studies. Moreover, as various resected organs entail different risks of morbidity, a weighed resected organ score system would help to standardize and adjust the details of radical resection. Third, the SEER database provided only a little information about the specific RPS classification system (e.g., French Federation of Cancer Centers Sarcoma (FNCLCC) or National Cancer Institute (NCI) grade), hence, we used the AJCC staging system to identify the patients with stage I RPS and the SEER database to find the tumor grades, which may omit biological information on mitotic activity and necrosis. Fourth, the details of the radiotherapy treatments were limited in the SEER database and the use of radiotherapy to treat RPS patients varied between the different centers. To date, it is still debated whether radiotherapy impacts the survival of patients with RPS and future randomized controlled trials are needed to provide a more precise conclusion. We await the results of the EORTC (European Organization for Research and Treatment of Cancer) study which compared the impact of surgery alone *versus* preoperative radiotherapy followed by surgery. Fifth, our study was retrospective in nature and subjective to potential innate bias similar to previously published studies.

## Conclusion

In the overall cohort of patients with stage I RPS, radical resections were not associated with improved midterm survival outcomes. However, radical resections might improve the overall survival of several subgroups of early-stage RPS patients (e.g., male patients or patients who received radiotherapy. Additionally, given the potential survival difference that we observed after eight years during the follow-up, more studies involving long-term surveillance are warranted in the future.

## Data Availability Statement

Publicly available datasets were analyzed in this study. This data can be found here: Surveillance, Epidemiology, and End Results (SEER) Program (https://seer.cancer.gov/about/overview.html).

## Ethics Statement

Ethical review and approval was not required for the study on human participants in accordance with the local legislation and institutional requirements. Written informed consent for participation was not required for this study in accordance with the national legislation and the institutional requirements.

## Author Contributions

CW & JW: Concept and design; data acquisition and analysis, interpretation of data; writing initial draft. JZ: Concept and design; data acquisition and analysis, interpretation of data; critical revision of the article. BH: Statistical analysis; interpretation of data; critical revision of the article. DY: Statistical analysis; interpretation of data; writing initial draft; and critical revision of the article. TW: Acquisition, analysis; interpretation of data; writing initial draft. All authors contributed to the article and approved the submitted version.

## Funding

This work was supported by the Sichuan Foundation of Science and Technology [grant number: 2020YFS0247].

## Conflict of Interest

The authors declare that the research was conducted in the absence of any commercial or financial relationships that could be construed as a potential conflict of interest.
