# Supplementary material for: Radical Versus Non-Radical Resection for Early-Stage Retroperitoneal Sarcoma: A Propensity Score-Matched Analysis
Source: Front Oncol. 2021 Jul 14;11:706543. doi: 10.3389/fonc.2021.706543 (PMC8316718; doi:10.3389/fonc.2021.706543)
Supplement: Supplementary file 1 [file Presentation_1.pdf]

Supplementary Material

1 Supplementary Data

1.1 Supplementary materials_1

Data-Use Agreement

Last Name: Weng
SEER ID: 10672-Nov2018
Request Type: Internet Access

SURVEILLANCE, EPIDEMIOLOGY, AND END RESULTS PROGRAM
Data-Use Agreement for the SEER 1975-2016 Research Database File

It is of utmost importance to protect the identities of cancer patients. Every effort has been made to exclude identifying information on individual patients from the computer files. Certain demographic information - such as sex, race, etc. - has been included for research purposes. All research results must be presented or published in a manner that ensures that no individual can be identified. In addition, there must be no attempt either to identify individuals from any computer file or to link with a computer file containing patient identifiers.

In order for the Surveillance, Epidemiology, and End Results Program to provide access to its Research Data File to you, it is necessary that you agree to the following provisions.

1. I will not use - or permit others to use - the data in any way other than for statistical reporting and analysis for research purposes. I must notify the SEER Program if I discover that there has been any other use of the data.
2. I will not present or publish data in which an individual patient can be identified. I will not publish any information on an individual patient, including any information generated on an individual case by the case listing session of SEER*Stat. In addition, I will avoid publication of statistics for very small groups.
3. I will not attempt either to link - or permit others to link - the data with individual level records in another database.
4. I will not attempt to learn the identity of any patient whose cancer data is contained in the supplied file(s).
5. If I inadvertently discover the identity of any patient, then (a) I will make no use of this knowledge, (b) I will notify the SEER Program of the incident, and (c) I will inform no one else of the discovered identity.
6. I will not either release - or permit others to release - the data - in full or in part - to any person except with the written approval of the SEER Program. In particular, all members of a research team who have access to the data must sign this data-use agreement.
7. I will use appropriate safeguards to prevent use or disclosure of the information other than as provided for by this data-use agreement. If accessing the data from a centralized location on a time sharing computer system or LAN with SEER*Stat or another statistical package, I will not share my logon name or password with any other individuals. I will also not allow any other individuals to use my computer account after I have logged on with my logon name and password.
8. For all software provided by the SEER Program, I will not copy it, distribute it, reverse engineer it, profit from its sale or use, or incorporate it in any other software system.
9. I will cite the source of information in all publications. The appropriate citation is associated with the data file used. (Please see either Suggested Citations on the SEER*Stat Help menu or the Readme.txt associated with the ASCII text version of the SEER data.)

My signature indicates that I agree to comply with the above stated provisions.

Chengxin Weng
Signature

2019.4.17
Date

Please print, sign, and date the agreement. Send the form to The SEER Program:

- **By fax to 301-680-9571**
- **Or, e-mail a scanned form to seerfax@imsweb.com**

Last Name: Weng | SEER ID: 10672-Nov2018 | Request Type: Internet Access

1.2 Supplementary materials_2

Details of variables of interest

Pathology Classification

The histology codes were referenced to confirm that the patients selected had common retroperitoneal sarcomas instead of those epithelial origin or those commonly occur in other space and not often within the retroperitoneal space. In addition, the histology codes were divided into four groups: well differentiated liposarcoma, Dedifferentiated liposarcoma, leiomyosarcoma, and other for the remaining histology codes, as the first three were the most common histologic types of retroperitoneal sarcomas.

Disease Stage

For the disease stage, both 6th and 7th AJCC staging systems were considered, the stages of 7th AJCC were used If there was a difference between the 6th and 7th editions.

Radiotherapy and Chemotherapy

Radiotherapy documented in SEER contained the radiotherapy sequence with surgery and radiotherapy types. No radiotherapy was received if the patient was described as ‘No radiotherapy and/or cancer-directed surgery’ in the ‘Radiotherapy sequence with surgery’ variable because the patient who did not undergo cancer-directed surgery were previously excluded.

Data of chemotherapy in SEER only include the chemotherapy recodes, and the ‘Unknown’ had been combined with ‘None’. So the chemotherapy recodes were only categorized as ‘chemotherapy’ and ‘None’ (no evidence of chemotherapy was found in the medical records examined).

Tumor size

Tumor size was selected as a covariate due to the Eighth edition of the AJCC Cancer Staging Manual expanding T classification into more than two criteria, and it was categorized as larger than 150(mm) or not for further analysis.

Tumor Grade

FNCLCC Grade criteria was not selected as a covariate due to incomplete patient records in our patient population. Instead, the Grade of SEER database were used in this study, which contains four categories: ‘Well differentiated; Grade I’, ‘Moderately differentiated; Grade II’, ‘Poorly differentiated; Grade III’, ‘Undifferentiated; anaplastic; Grade IV’, ‘Unknown’. Patients with unknown or missing values in above variables except radiotherapy and chemotherapy were excluded.

2 Supplementary Figures and Tables

2.1 Supplementary Figures

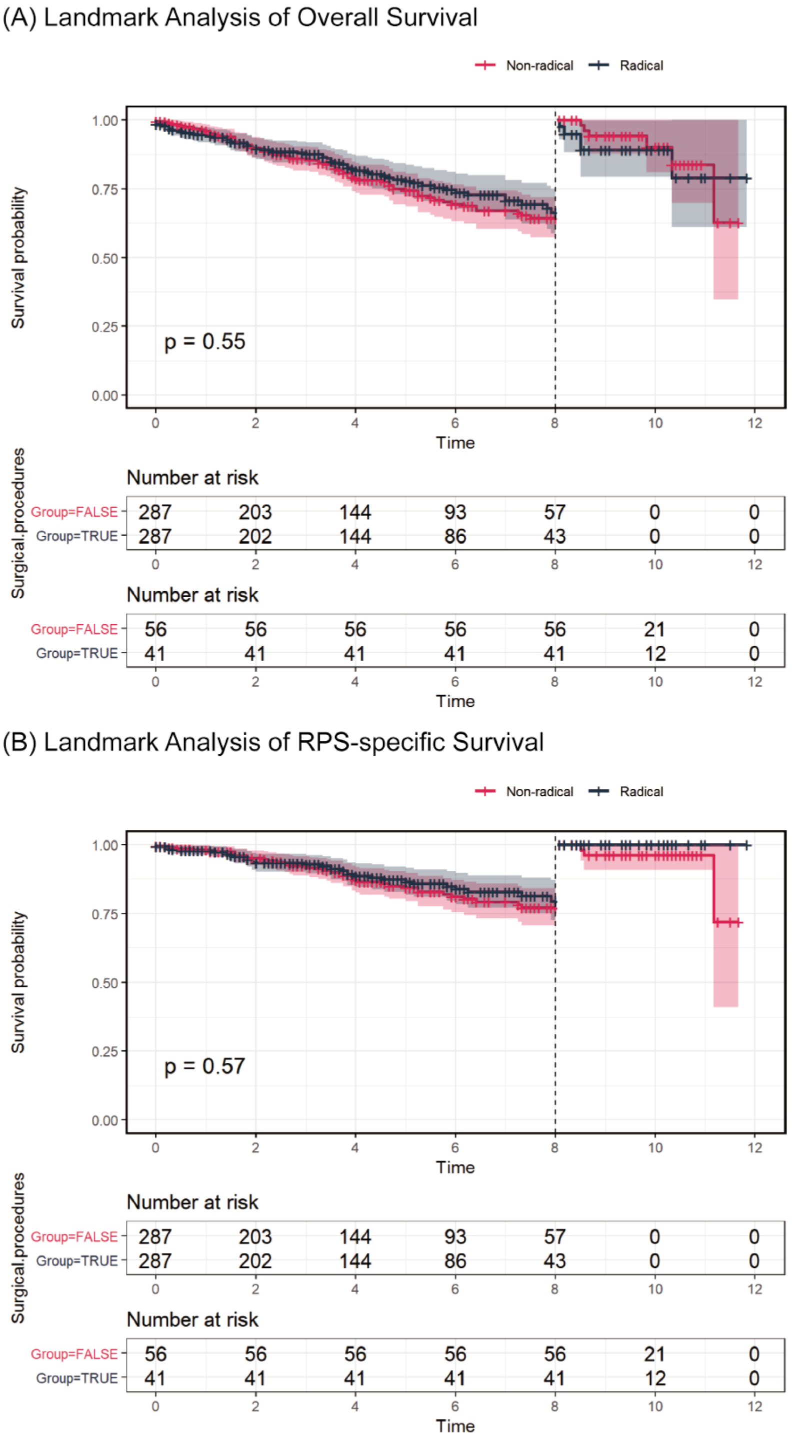

Supplementary Figure 1 Landmark Analysis of survival outcomes for Stage I Retroperitoneal Sarcoma Patients. (A) landmark analysis for overall survival; (B) landmark analysis for RPS-specific survival.

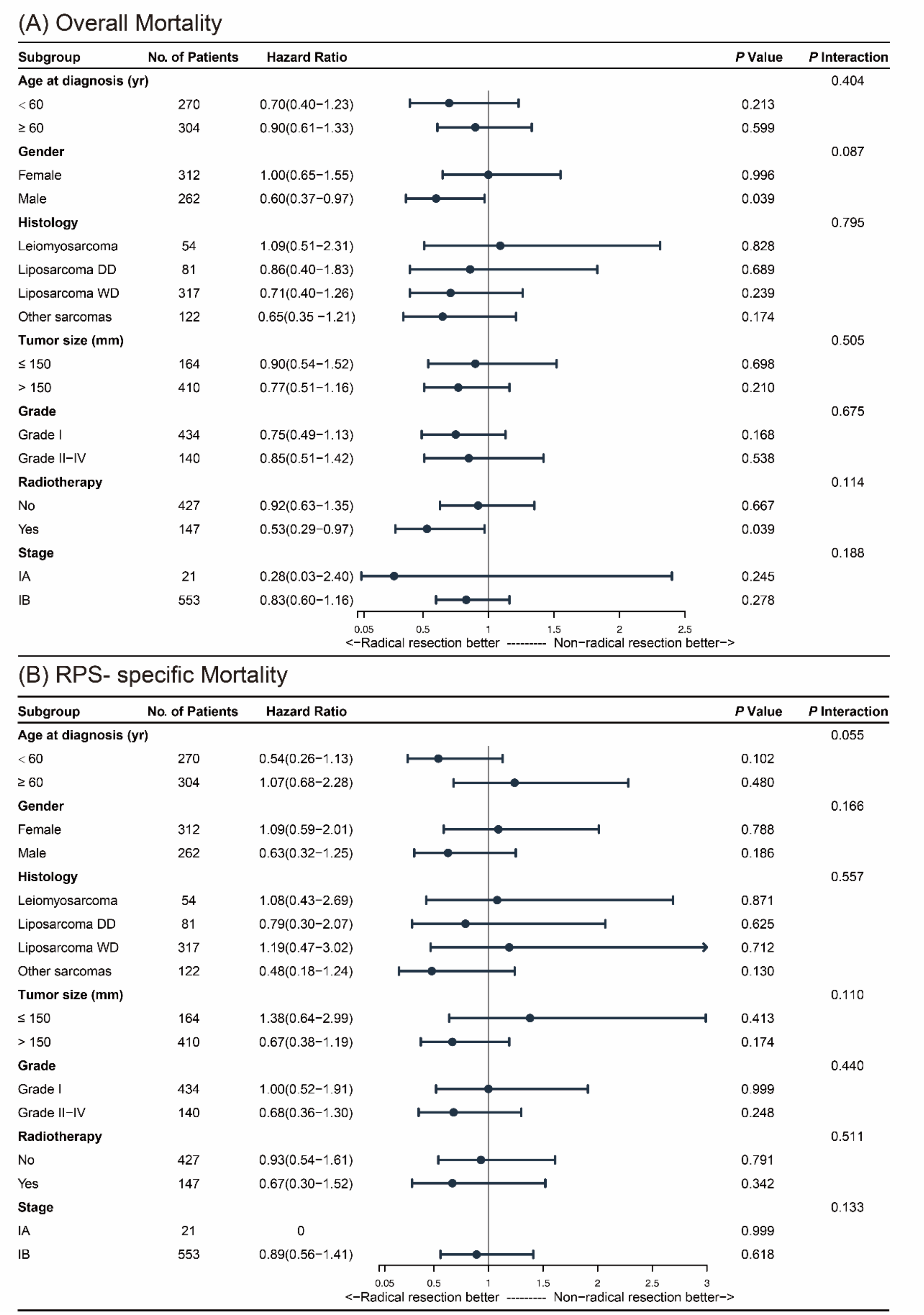

Supplementary Figure 2 Subgroup analyses after propensity score matching for stage I retroperitoneal sarcoma (RPS) patients. (A) subgroup analysis for overall mortality; (B) subgroup analysis for RPS-specific mortality.

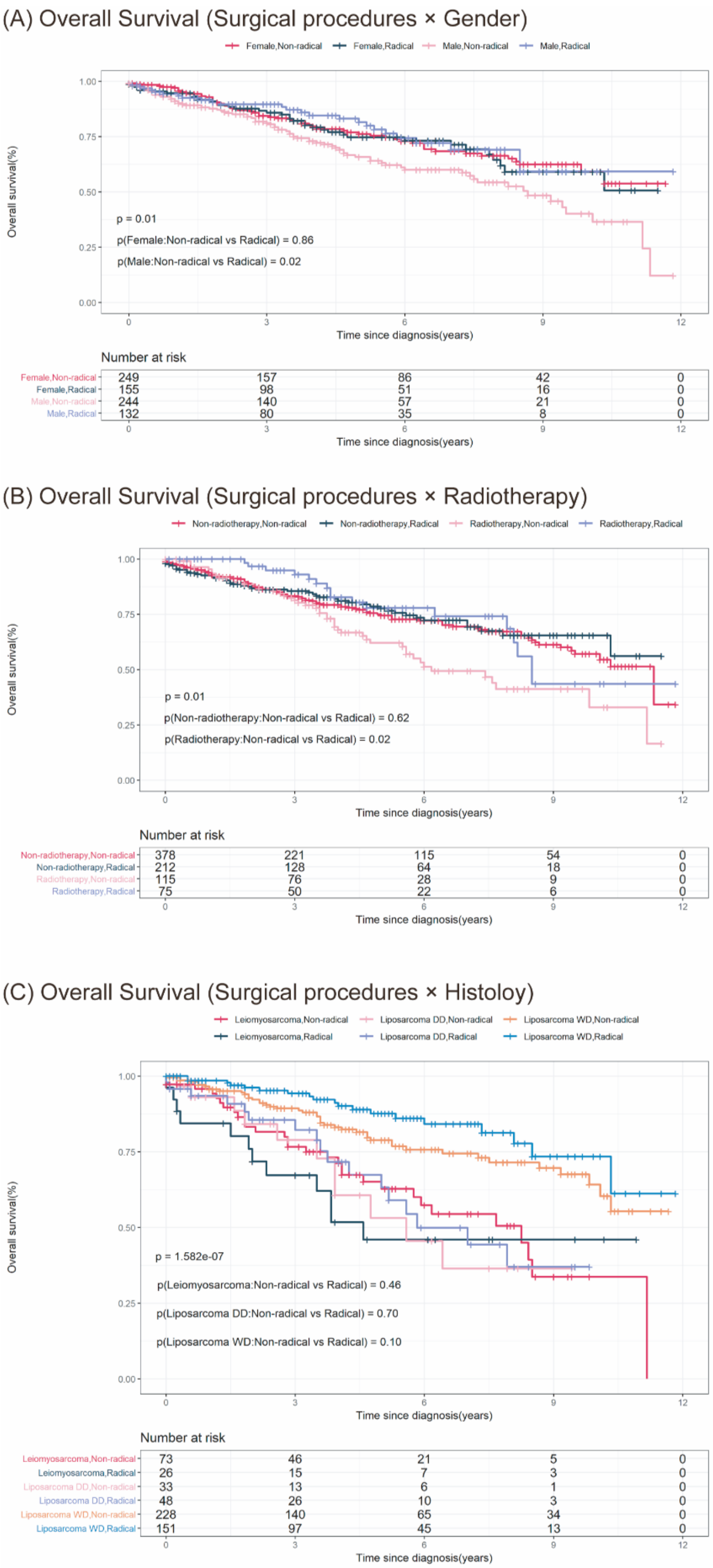

Supplementary Figure 3 Kaplan–Meier Curves of overall survival for stage I retroperitoneal sarcoma (RPS) patients stratified by different subgroups. (A) stratified by gender subgroups; (B) stratified by radiotherapy subgroups; (C) stratified by histological types.
